# Prevalence of Motion Sickness Among Saudi Residents: An Interview-Based Cross-Sectional Study

**DOI:** 10.3390/healthcare13222907

**Published:** 2025-11-14

**Authors:** Mahdi Mohammed Alturaiki, Hashim Radhi Alwayel, Hamad Mohammed Aldeen, Mahdi Aqeel AlmohammedAli, Hani Ali Alhabdan, Ahmed Mohammed Abuali, Abdullah Almaqhawi

**Affiliations:** 1 College of Medicine, King Faisal University, Al-Ahsa 31982, Saudi Arabia; 221417966@student.kfu.edu.sa (M.M.A.); 221405113@student.kfu.edu.sa (H.R.A.); 221434236@student.kfu.edu.sa (H.M.A.); 221418674@student.kfu.edu.sa (M.A.A.); 221419040@student.kfu.edu.sa (H.A.A.); 221409924@student.kfu.edu.sa (A.M.A.); 2 Department of Family and Community Medicine, College of Medicine, King Faisal University, Al-Ahsa 31982, Saudi Arabia

**Keywords:** motion sickness, dizziness, vestibular, prevalence, metro commuters, public transportation

## Abstract

**Background/Objectives**: Motion sickness is a prevalent neuro-vestibular syndrome that affects individuals across various modes of transport and can significantly impact quality of life and travel safety. This study aimed to assess the prevalence, severity, and associated risk factors of severe dizziness related to motion sickness among adult residents in the Kingdom of Saudi Arabia (KSA), with particular focus on socio-demographic and behavioral determinants. **Methods**: A cross-sectional survey was conducted among 349 participants recruited primarily from the Riyadh region. A structured questionnaire captured demographic variables, personal health history, and experiences of dizziness and related symptoms during air or metro travel. Chi-square tests and multivariable logistic regression were applied to examine associations between dizziness and potential predictors, with *p* ≤ 0.05 considered significant. **Results**: Overall, (23.5%) of respondents reported experiencing severe dizziness during metro travel (82/349). Females were more affected than males (32.1% vs. 15.8%; χ^2^(1) = 12.06, *p* = 0.0005, Cramer’s V = 0.186), although this association lost significance in the adjusted model. Height showed a borderline association (*p* = 0.053). In multivariable analysis, previous similar episodes were the strongest independent predictor of dizziness (aOR 15.63, 95% CI 6.40–38.16, *p* < 0.001). ANOVA revealed no difference in severity by sex or height (*p* > 0.7). **Conclusions**: Motion sickness affects nearly one-quarter of Saudi metro travelers (23.5%) and is predominantly influenced by a history of previous similar episodes rather than demographic factors. These findings underscore the need for targeted preventive strategies, ergonomic vehicle design, and public health education to mitigate the burden of motion sickness in the KSA’s expanding transportation systems.

## 1. Introduction

Motion sickness (MS) is triggered by discordance between visual, vestibular, and proprioceptive inputs and may present with nausea, dizziness, vomiting, and autonomic symptoms. Epidemiologically, MS is not uncommon. A 2023 meta-analysis of population surveys found that between 45% and 74% of adults worldwide experience at least one episode over their lifetime, depending on the definition used and the mode of transport examined [1]. Incidence peaks during childhood (ages 6–12 years); dips in adolescence; rises again during the reproductive years, especially in women; and declines after the sixth decade [2]. Such patterns imply biological as well as behavioral determinants. The constellation of recognized risk factors includes female sex, younger age, migraine, inner-ear pathology, poor sleep, anxiety, and fasting prior to travel [3]. At the population level, these determinants skew the global burden towards women and coastal or island communities with high ferry use.

Physiological and genetic susceptibilities intersect to modulate individual thresholds. A Medscape clinical review summarizing decades of laboratory and field work underscores the influence of hormonal cycles, vestibular sensitivity, and concurrent medication on symptom propensity [4]. Twin-study data reinforce the biological underpinnings; monozygotic pairs show concordance far exceeding that of dizygotic twins, with heritability estimates hovering around 55–70%, and the genetic signal being strongest in childhood [5]. Polymorphisms in HTR3B, RYR2, and genes governing calcium signaling have all been implicated, though no single locus exerts a major effect.

The characteristics of motion itself—frequency, amplitude, direction, predictability—are equally decisive. A 2023 systematic review and meta-analysis of in-vehicle studies reported that oscillations below 0.3 Hz, rear-facing or lateral seating, and frequent screen use amplified nausea scores up to threefold [6]. With the advent of autonomous vehicles and electric cars—whose instantaneous torque and regenerative braking create unique motion profiles—engineers are racing to devise ride-quality algorithms that minimize provocative frequencies. Meanwhile, the digital revolution has led to a new development: visually induced motion sickness (VIMS), often labeled cybersickness. A 2024 PRISMA-guided review of virtual-reality (VR) studies spanning medicine, education, and entertainment found that up to 80% of naïve users develop symptoms within 15 min of immersion; field-of-view, frame lag, and scene complexity were dominant predictors [7]. As VR and augmented-reality platforms permeate classrooms and workplaces, VIMS is poised to rival classic travel sickness in prevalence.

Beyond transient discomfort, MS carries tangible economic costs. The U.S. Navy has long regarded airsickness as a formidable obstacle to flight training; a 2001 aerospace medicine report estimated that one training air wing incurred more than USD 150,000 annually in extra fuel and maintenance because of sick sorties and remedial flights [8]. In commercial sectors, crew absenteeism, tourist cancellations, and negative customer reviews translate into millions in lost productivity. The aerospace industry, therefore, invests heavily in simulator design, adaptation protocols, and pharmacological countermeasures—not merely to preserve comfort but to safeguard operational readiness and profit margins.

In the Kingdom of Saudi Arabia (KSA), several converging trends magnify the relevance of motion-related malaise. The government’s Vision 2030 has spurred an expansion of high-speed rail, a multi-line Riyadh Metro, and a surge in domestic air travel, exposing residents to a broader array of motion environments than ever before. Smart-phone penetration tops 95%, and VR gaming cafés have multiplied in major cities. Local health authorities in Saudi Arabia provide publicly accessible first-aid guidance for travel-related nausea and vertigo (for example via the Ministry of Health – Saudi Arabia First-Aid portal) that includes recommendations such as head support and avoiding reading during motion [9]. Yet peer-reviewed prevalence data from the KSA remain strikingly scarce, limited to small occupational cohorts or naval cadets. Without such baseline figures, clinicians cannot benchmark interventions, public-health educators cannot tailor messaging, and transport engineers cannot quantify the impact of design changes.

Accordingly, we undertook what is, to our knowledge, the first nationwide community survey of motion sickness among Saudi residents. By combining a validated susceptibility instrument with detailed socio-demographic and behavioral questions, our study aimed not only to establish prevalence but also to highlight modifiable risk factors. These data will help stakeholders—from urban-mobility planners to primary-care physicians—to develop evidence-based strategies that reduce morbidity, improve travel experience, and bolster the Kingdom’s broader goals of healthy, connected communities. This study aimed to determine the prevalence of motion sickness symptoms among Saudi residents. Secondary objectives included assessing associations between motion sickness and socio-demographic characteristics (age, sex, height, weight, education, and marital status) and exploring behavioral factors such as travel frequency, device use during motion, recent illness, and chronic diseases that may affect symptom severity.

## 2. Materials and Methods

### 2.1. Study Design

A descriptive, analytical, cross-sectional study was carried out between January and March 2025.

### 2.2. Study Setting, Participants, Recruitment, and Sampling

The study targeted residents of all 13 administrative regions of Saudi Arabia, KSA. Study information was explained to each participant, and informed consent was obtained verbally before initiating the interview. Eligibility criteria included Arabic or English literacy, age ≥ 16 years (with consent obtained from legal guardians for minors), and current residence in the Kingdom for at least six months. Visitors, individuals with impaired consciousness, and those unable to give informed consent were excluded. Ethical approval was obtained in compliance with the Helsinki Declaration.

The study was conducted in the Riyadh Metro, the official rapid transit system in Riyadh, Saudi Arabia. All surveys were conducted at the King Abdullah Financial District (KAFD) station, as it is the major hub in the metro network that connects multiple lines. Participants were recruited systematically from metro cabins at varying times of the day to ensure a diversity of responses. From each cabin, a sample of 2–5 individuals was randomly selected and invited to participate.

Data were collected using a face-to-face, interviewer-administered questionnaire lasting between 15 and 25 min. Participants were provided with the standardized questionnaire items, and the researcher clarified questions or answered inquiries when needed, but responses were recorded directly by the participants themselves.

To assess motion sickness susceptibility, selected items were incorporated from the validated Motion Sickness Susceptibility Questionnaire–Short form (MSSQ-Short), originally developed by Golding (2006) [10]. The selected items included questions assessing dizziness or nausea while traveling by car, bus, train, aircraft, or ship, as these represent the most common motion environments.

A non-probability intercept sample was obtained by randomly selecting individuals exiting different stations of the Riyadh Metro and inviting them to participate in short interviews. Using n = z^2^ p(1 − p)/d^2^ and assuming *p* = 0.5 (maximum variability), precision d = 0.05, and Z = 1.96, the minimum required sample was 384. Allowing for incomplete responses (15%), the target size was 450. A total of 349 complete responses (77.6% of target) were available for analysis. The research was conducted in conformance with the guidelines outlined in the Strengthening the Reporting of Observational Studies in Epidemiology (STROBE) Statement: guidelines for reporting observational studies (Table S1).

### 2.3. Data Collection Instrument

The questionnaire comprised three sections:

1. Sociodemographic questions (8 items).
2. Developed criteria for this study: Dizziness-related diseases, chronic conditions.
3. Motion sickness experience: Adapted items from the MSSQ-Short [10], combined with severity scales.

The questionnaire consisted of three components. The first section included eight items on biographical data (e.g., age, gender, and academic background) to provide contextual information for analysis. The second section comprised items specifically developed for this study to address the research objectives; these items were reviewed for clarity and content validity by two consultants from different specialties and were refined following a pilot study with (n = 30) demonstrated acceptable internal consistency (Cronbach’s α = 0.89). The final section incorporated selected items from MSSQ-Short [10], with only the relevant subscales adopted to maintain focus and reduce respondent burden. The resulting instrument thus combined demographic information, researcher-developed items, and adapted standardized measures to ensure both contextual relevance and methodological rigor (Table A1).

### 2.4. Data Entry and Analysis

Data were exported from Google Forms to Microsoft Excel, cleaned and coded, and then analyzed with IBM SPSS v26. Descriptive statistics (means ± SD for continuous variables and frequencies/percentages for categorical variables) summarized the dataset. Associations between categorical variables and the presence of severe motion sickness-related dizziness were examined using Pearson’s χ^2^ test or Fisher’s exact test as appropriate, while independent-samples t-tests and one-way ANOVA were used for continuous comparisons when applicable. A multivariable logistic regression model was employed to identify independent predictors of severe dizziness, and adjusted odds ratios (aORs) with 95% confidence intervals (CIs) were reported. All tests were two-tailed, and a *p* ≤ 0.05 was considered statistically significant.

## 3. Results

Table 1 displays various demographic parameters of the participants, with a total number of 349. The mean age was 29.7 ± 8.3 years, ranging from less than 18 to over 45 years. Most of the participants were aged 22–45 years (61.0%), whereas those less than 18 years constituted the lowest percentage (2.6%). The gender breakdown was fairly balanced between 47.3 percent female to 52.7 percent male. Amongst the female participants, there was a low percentage of women who were pregnant (0.6%). In terms of height, it was quite evenly distributed, with 34.1 percent measuring 160 cm and below. The same tendency was found with weight, with 34.7% of participants weighing 60 kg and under. A large percentage (75.1) of the respondents are of Saudi nationality and the majority (69.6) of them are based in the Riyadh region. The level of education was also very high, with 76.5 percent having attained at least a bachelor’s degree. Most of the respondents were single (65.9%).

**Figure 1:**
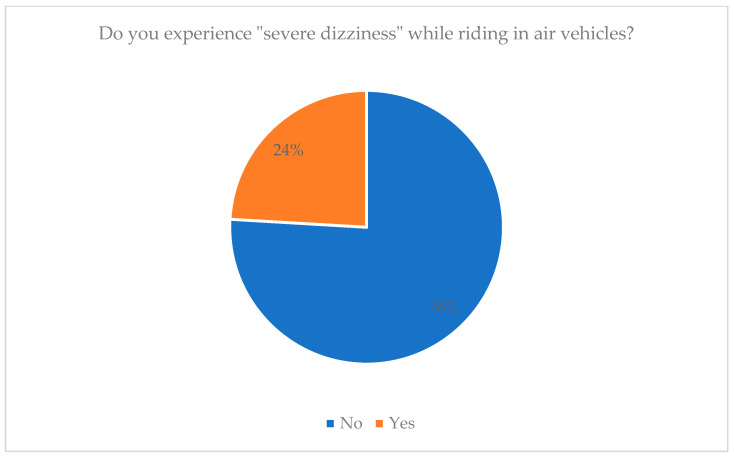
Illustrates experiencing severe dizziness while riding in air vehicles among participants.

As shown in Figure 1, the results indicate that 75.9% (265) of the 349 respondents stated that they do not experience severe dizziness when traveling in air vehicles, giving the following specific responses: 265 No. On the other hand, 84 people (24.1%) responded that they do suffer some form of severe dizziness in these circumstances.

As illustrated in Table 2, the results of the personal history survey (n = 349) highlight several important issues about motion sickness. Approximately 84% of participants did not report any disease-causing dizziness; among the 16% who did, anemia was the most common condition (52.6% of that subgroup). In the case of air travel, 24.1 percent feel extremely dizzy. Out of them, the majority had mild (40.5%) to moderate (42.9%) severity. Dizziness was followed closely by nausea, which was its most common additional effect (88.7%). Only 5.2% reported fear or anxiety about riding in moving vehicles. With respect to headache, 73.1 per cent had no headache, 17.2 per cent had a generalized character of headache, and 8.3 per cent had migraine. Lastly, 84.8 percent of the respondents had no chronic diseases.

**Figure 2:**
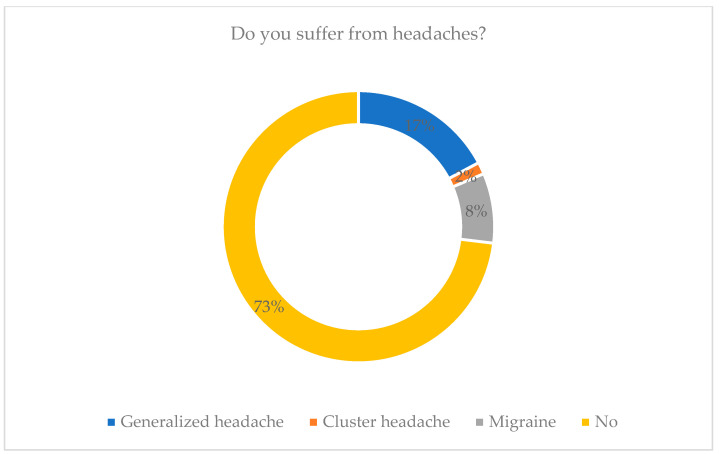
Illustrates experiencing extreme headaches while riding in vehicles among participants.

As shown in Figure 2, the data presented illustrate the prevalence of different types of headaches among the study population. The most prevalent type was generalized headache, occurring in 60% of the participants. Migraine headaches also constitute a significant percentage, affecting 29 percent of people. Cluster headaches are less reported and make up 5% of the sample. Intriguingly, 255 of them said that they did not experience any headaches.

The statistics presented in Table 3 (n = 349) show that there could be a number of predisposing factors to motion sickness among respondents. A small proportion of participants (13.2%) stated that they had an illness in the days beforehand, with most of those illnesses being the flu (86.9 percent of those with infection). Moreover, 22.1 percent used medication regularly, most commonly supplements (20.8 percent among those using medication). In addition, 85.4 percent of participants used the metro more than three times a week. During the commute, most passengers used their phones (64.5%). Only a few participants reported feeling unwell on their way to work (nausea, 8.0%; dizziness, 4.9%), whereas most felt nothing. The majority of the passengers reported feeling OK throughout their transit (85.7%), though a portion of them were slightly annoyed (13.8%). Lastly, most respondents (74.0%) stated that they experienced such symptoms earlier, and most of them lacked anything to eat to feel better (85.7%).

Table 4 shows that suffering from dizziness or vertigo has a statistically significant relation to gender (*p* value = 0.0005) and a borderline, non-significant relation to height (*p* value = 0.053). It also shows a statistically insignificant relation to age, weight, nationality, educational level, and marital status. Female gender was statistically significantly associated with the presence of diseases causing dizziness or vertigo, while height did not show a significant association after adjustment.

Table 5 shows that having a fear or anxiety about riding in moving vehicles has a statistically significant relation to educational level (*p*-value = 0.001). It also shows a statistically insignificant relation to gender, age, weight, nationality, height, and marital status. Educational level was significantly associated with fear or anxiety about riding in moving vehicles (*p* = 0.001). Participants with lower educational levels (particularly those with a high school education or lower) were more likely to report fear or anxiety compared with those with a bachelor’s degree or higher.

## 4. Discussion

Motion sickness remains a clinically important yet incompletely understood neuro-vestibular phenomenon. The present cross-sectional study set out to quantify the prevalence and correlates of self-reported severe dizziness during mass transit use among residents of the Kingdom of Saudi Arabia (KSA), thereby filling a recognized epidemiological gap. By surveying 349 adults drawn largely from the Riyadh metropolitan region, we found that 23.5% experienced “severe dizziness” during metro travel, and that those of female sex showed a significant unadjusted association (*p* = 0.0005) but lost significance after adjustment, while height demonstrated a borderline relationship (*p* = 0.053). Previous episodes were the strongest independent predictor (aOR 15.63, 95% CI 6.40–38.16, *p* < 0.001). Globally, lifetime exposure to motion sickness symptoms spans a wide range. A 2023 Indian community survey that used the same Motion-Sickness Susceptibility Questionnaire (MSSQ) reported a point prevalence of 75% for at least one symptomatic episode, with 27% describing incapacitating nausea or vertigo [1]. Our more restrictive case definition (“severe dizziness” rather than any symptom) almost certainly explains why the proportion we observed is lower. Nevertheless, the finding that nearly one-quarter of Saudi metro commuters experience severe dizziness underscores a substantial and previously undocumented national burden.

Vehicle-specific factors amplify this burden. A recent meta-analysis of 57 experimental studies demonstrated that low-frequency oscillations (<0.3 Hz), rear-facing seating, and non-driving tasks such as smartphone use increase motion sickness odds by up to threefold [11]. Our dataset echoes these mechanistic insights; a total of 64.5% of participants browsed their phones in transit, and this subgroup contributed disproportionately to nausea co-symptoms. In the KSA, where high-speed rail and autonomous-capable electric vehicles are expanding rapidly, design strategies that dampen very-low-frequency sway and integrate anticipatory cues may therefore be pivotal.

Sex differences emerged clearly; although females showed higher rates of dizziness (32.1% vs. 15.8%) in unadjusted analyses, this effect was no longer significant after controlling for other variables. Laboratory work using opto-kinetic drum paradigms finds a similarly higher symptom load in females, even when vestibular thresholds are matched [12]. Hormonal modulation of otolith afferent gain and slower habituation responses are hypothesized mediators, while pregnancy adds transient vulnerability via altered autonomic tone. Our survey captured only one pregnant traveler, but the pattern aligns with sex steroid influences suggested in the broader literature.

Age effects were less pronounced, yet a non-significant trend towards greater dizziness in those > 45 years was noted. A 2023 systematic review of individual predictors confirmed that advancing age generally reduces motion sickness susceptibility, likely through vestibular hypofunction, but highlighted substantial heterogeneity between 40 and 60 years [13]. The near-flat age gradient in our data may thus reflect countervailing factors such as comorbid chronic disease, polypharmacy, and reduced sleep, all common in mid-life Saudi adults.

Comorbidity with migraine is well established. In our sample, 8.3% reported migraine headaches, and migraineurs were over-represented among those with severe dizziness. Abouzari and colleagues demonstrated that vestibular migraine co-exists with high MSSQ scores in up to 50% of susceptible university students, with shared genetic loci (e.g., PRDM16 (PR Domain Zinc Finger Protein 16)) suspected [14]. Although we did not genotype participants, the overlap strengthens the argument for proactive screening of migraine patients who report travel-related nausea, as vestibular suppressants or triptan prophylaxis may provide dual benefits.

A previously observed but statistically non-significant result was the association between short stature and dizziness (*p* = 0.053) (32.1% vs. 15.8% across height groups). Height is rarely examined, but finite-element head–neck models predict larger rotational acceleration at the labyrinth in shorter individuals seated on the same suspension plane. The vehicle meta-analysis cited above noted an effect size of d = 0.34 for stature but deemed the evidence to be of low certainty [11]. Although the relationship in our study did not reach statistical significance, the trend suggests that anthropometric considerations could inform seatbelt anchorage and headrest heights in regional rail rolling stock.

Behavioral modifiers were equally salient. Most participants traveled after a latency of ≥4 h post-meal and 85.7% ingested nothing en route, practices shown to exacerbate gastric dysrhythmia. Concurrent screen exposure links the classic vestibular–ocular conflict model with visually induced motion sickness (VIMS). A 2024 PRISMA-guided review of VR interventions found disorientation and nausea in 63% of head-mounted display users, with large viewing angles and high scene complexity as predictors [7]. Smartphone video viewing in a moving metro yields an analogous visual–vestibular mismatch and likely accounts for the high prevalence of associated nausea (88.7%) we observed.

From a therapeutic standpoint, evidence for pharmacological prophylaxis remains limited. A 2022 Cochrane meta-analysis concluded that first-generation antihistamines reduce symptom risk relative to placebo (RR 1.81, 95% CI 1.23–2.66), but at the cost of sedation, and high-quality pediatric data were absent [15]. Given that 77.9% of our respondents used no long-term medication, and only 5.2% reported travel anxiety, non-pharmacological strategies (horizon fixation, leaning headrest, limiting screen time) may yield substantial gains without side effects.

Emerging mechanistic work also emphasizes central adaptation. Golding and co-workers recently showed that a single session of provocative physical motion sensitizes individuals to subsequent visually induced motion sickness, implying a shared higher-order accumulation filter in the brainstem–cerebellar network [16]. This resonates with our observation that 74% of symptomatic passengers had experienced similar episodes previously, supporting our regression finding that prior episodes were the strongest independent predictor (aOR 15.63, 95% CI 6.40–38.16, *p* < 0.001).

Several limitations must temper interpretation. Firstly, although the study was conducted as face-to-face interviews in the Riyadh Metro—an approach that reduces the bias risk associated with online convenience sampling—it still primarily reflects the experiences of metro commuters in an urban setting, which may limit generalizability to rural residents or those who do not frequently use public transportation. Secondly, our reliance on self-report introduces recall and social-desirability bias; objective physiological markers (e.g., electrogastrography, vestibular function tests) were not obtained. Thirdly, the cross-sectional design cannot resolve temporal sequences: for example, whether phone use precipitates symptoms or users reach for their phones as a distraction once dizziness begins. Fourthly, although we identified significant associations, residual confounding (sleep deprivation, menstrual cycle, seat location) may persist. Finally, the study fell short of its a priori sample size target, potentially under-powering analyses of rare exposures such as pregnancy. Moreover, it should be considered that the observed effects of screen engagement during transit may have been influenced by screen size, as variations in display dimensions could modulate visual field involvement and vestibular stimulation, thereby affecting susceptibility to motion-related discomfort [17]. Furthermore, participants’ height and weight were self-reported, which may have led to minor inaccuracies in anthropometric data due to recall or reporting bias.

## 5. Conclusions

The present survey quantifies a 23.5% prevalence of extreme motion-related dizziness among Saudi travelers and demonstrates that previous similar episodes were the strongest independent predictor (aOR 15.63, 95% CI 6.40–38.16, *p* < 0.001), while female sex and short stature showed only unadjusted or borderline associations. These insights dovetail with international literature and underscore the need for ergonomically optimized vehicle interiors, targeted public-health messaging, and integrative management protocols that span lifestyle advice to evidence-based pharmacology. Future longitudinal cohorts enriched for physiological and genomic markers will be critical to unravel causal pathways and to personalize counter-measures in the rapidly evolving mobility landscape of the KSA.

## Figures and Tables

**Table 1:** Sociodemographic characteristics of participants (n = 349).

Parameter		No.	Percent (%)
Age	Less than 18	9	2.6
	18 to 21	95	27.2
	22 to 45	213	61
	More than 45	32	9.2
Gender	Female	165	47.3
	Male	184	52.7
Pregnant female (n = 165)	Yes	1	0.6
	No	164	99.4
Height (Mean = 166.5, SD = 10.3)	160 cm or less	119	34.1
	161 to 170	108	30.9
	More than 170 cm	122	35
Weight (Mean = 70.4, SD = 19.3)	60 kg or less	121	34.7
	61 to 75	111	31.8
	76 kg or more	117	33.5
Nationality	No	87	24.9
	Yes	262	75.1
Region of residence	Riyadh	243	69.6
	Eastern region	31	8.9
	Qassim	7	2
	Madinah	5	1.4
	Jazan	12	3.4
	Makkah	6	1.7
	Baha	4	1.1
	Others	41	11.7
Educational level	Primary level	2	0.6
	Middle school	10	2.9
	High school	69	19.8
	Bachelor’s or higher	267	76.5
	Illiterate	1	0.3
Marital status	Single	230	65.9
	Married	114	32.7
	Divorced or widowed	5	1.4

**Table 2:** Parameters related to personal history regarding motion sickness (n = 349).

Parameter		No.	Percent (%)
Do you suffer from any disease that causes dizziness or vertigo?	No	292	83.7
	Yes	57	16.3
If yes, please state the disease. (n = 57)	Anemia	30	52.6
	BPPV	1	1.8
	Bronchial asthma	2	3.5
	Hypoglycemia	7	12.3
	Hypotension	3	5.3
	Otitis media	4	7
	Thyroid disorders	2	3.5
	Tumor	1	1.8
	Vitamin D deficiency	2	3.5
	Unknown	4	7
Do you experience “severe dizziness” while riding in vehicles?	No	265	75.9
	Yes	84	24.1
If yes, how severe is the dizziness or vertigo? (n = 84)	Mild	34	40.5
	Moderate	36	42.9
	Severe	11	13.1
	Unbearable	3	3.6
Do you experience other effects in addition to dizziness or light-headedness? (n = 71)	Nausea	63	88.7
	Dizziness	1	1.4
	Stomachache	23	32.4
	Vomiting	17	23.9
	Fainting	1	1.4
	Headache	1	1.4
Do you have a fear or anxiety about riding in moving vehicles?	No	331	94.8
	Yes	18	5.2
Do you suffer from headaches?	Generalized headache	60	17.2
	Cluster headache	5	1.4
	Migraine	29	8.3
	No	255	73.1
Do you suffer from any chronic diseases?	Asthma	16	4.6
	Bipolar	1	0.3
	Hypertension	15	4.3
	Anemia	8	2.3
	Sinusitis	4	1.1
	Diabetes	19	5.4
	Epilepsy	1	0.3
	Hypothyroidism	1	0.3
	None	296	84.8

**Table 3:** Participants’ possible causative factors regarding motion sickness (n = 349).

Parameter		No.	Percent (%)
Have you had an infection in the past few days?	No	303	86.8
	Yes	46	13.2
If yes, please state the type of infection. (n = 46)	Flu	40	86.9
	Sinusitis	1	2.2
	Conjunctivitis	1	2.2
	Gastroenteritis	4	8.7
Do you use any medication on a regular basis?	No	272	77.9
	Yes	77	22.1
If yes, please state the medication or the reason for using it. (n = 77)	Analgesics	6	7.8
	Anti-convulsant	2	2.6
	Anti-hypertension drugs	8	10.4
	Asthma medications	8	10.4
	Diabetes medications	12	15.6
	Eye drops	2	2.6
	PPI	4	5.2
	Supplements	16	20.8
	Thyroxine	3	3.9
	Colon medications	15	2.6
	Others	23	19.5
How often do you use the metro?	This is the first time	28	6.6
	Two or three times	298	8
	More than three times	233	85.4
How do you spend your time in the subway?	Relaxing	33	
	Talking	24	9.5
	Moving and organizing	1	6.9
	Reading	21	0.3
	Looking out the window	36	6
	Checking the phone	1	10.3
Did you feel any of the following in the commute?	Surfing my phone	225	0.3
	Laptop	1	64.5
	Sleeping	2	0.3
	Praying	1	0.6
	Nothing	4	0.3
	Sweating	6	1.1
	Dizziness	17	1.7
	Nausea	28	4.9
	Abdominal pain	5	8
	Tired	8	1.4
	Fever	6	2.3
	Sleepy	16	1.7
	Unbalanced	2	4.6
	Others	6	0.6
	Nothing	291	1.7
How do you feel in general during the flight?	Fine	299	83.4
	Like I’m sick	2	85.7
	A bit upset	48	0.6
If you feel upset or sick, when did you feel that way? (n = 50)			13.8
	Start of boarding the metro	12	24
	Start of the metro’s movement	18	36
	When the trip ends	4	8
	Midway through the trip	16	32
Has this happened before? (n = 50)	No	13	26
	Yes	37	74
Does it get worse when you face the window? (n = 50)	No	37	74
	Yes	13	26
Does it get worse when you see that you are moving in the opposite direction? (n = 50)	No	33	66
	Yes	17	34
Did you eat anything to feel better before or during your travels?	Coffee	12	3.4
	Water	5	1.4
	Gum	29	8.3
	Ginger drink	2	0.6
	Others	2	0.6
	Nothing	299	85.7
When was the last meal you ate?	During the flight	4	1.1
	Shortly before the flight	73	20.9
	Two to four hours before the flight	87	24.9
	More than four hours	185	53
What kind was it?	Sweets	6	1.7
	Snack	119	34.1
	Complete Meal	224	64.2

**Table 4:** Relation between suffering from dizziness or vertigo and sociodemographic characteristics.

Parameter		Do You Suffer from any Disease that Causes Dizziness or Vertigo?		Total (n = 349)	*p* Value *	Adjusted *p* Value
		No	Yes			
Gender	Female	123	42	165	0.0001	0.087
		42.1%	73.7%	47.3%		
	Male	169	15	184		
		57.9%	26.3%	52.7%		
Age	Less than 18	6	3	9	0.075	
		2.1%	5.3%	2.6%		
	18 to 21	78	17	95		
		26.7%	29.8%	27.2%		
	22 to 45	185	28	213		
		63.4%	49.1%	61.0%		
	More than 45	23	9	32		
		7.9%	15.8%	9.2%		
Height	160 cm or less	89	30	119	0.003	0.053
		30.5%	52.6%	34.1%		
	161 cm to 170 cm	92	16	108		
		31.5%	28.1%	30.9%		
	More than 170 cm	111	11	122		
		38.0%	19.3%	35.0%		
Weight	60 kg or less	96	25	121	0.132	
		32.9%	43.9%	34.7%		
	61 kg–75 kg	92	19	111		
		31.5%	33.3%	31.8%		
	76 kg or more	104	13	117		
		35.6%	22.8%	33.5%		
Nationality	Saudi	215	47	262	0.159	
		73.6%	82.5%	75.1%		
	Non-Saudi	77	10	87		
		26.4%	17.5%	24.9%		
Educational level	Primary school	1	1	2	0.438	
		0.3%	1.8%	0.6%		
	Middle school	7	3	10		
		2.4%	5.3%	2.9%		
	High school	60	9	69		
		20.5%	15.8%	19.8%		
	Bachelor’s or higher	223	44	267		
		76.4%	77.2%	76.5%		
	Illiterate	1	0	1		
		0.3%	0.0%	0.3%		
Marital status	Single	191	39	230	0.587	
		65.4%	68.4%	65.9%		
	Married	96	18	114		
		32.9%	31.6%	32.7%		
	Divorced or widowed	5	0	5		
		1.7%	0.0%	1.4%		

* *p* value was considered significant if ≤0.05.

**Table 5:** Riding in moving vehicles—association with sociodemographic characteristics.

Parameter		Do You Have Fear or Anxiety About Riding in Moving Vehicles?		Total (n = 349)	*p* Value *
		No	Yes		
Gender	Female	154	11	165	0.227
		46.50%	61.10%	47.30%	
	Male	177	7	184	
		53.50%	38.90%	52.70%	
Age	Less than 18	8	1	9	0.522
		2.40%	5.60%	2.60%	
	18 to 21	88	7	95	
		26.60%	38.90%	27.20%	
	22 to 45	204	9	213	
		61.60%	50.00%	61.00%	
	More than 45	31	1	32	
		9.40%	5.60%	9.20%	
Height	160 cm or less	110	9	119	0.341
		33.20%	50.00%	34.10%	
	161 cm to 170 cm	104	4	108	
		31.40%	22.20%	30.90%	
	More than 170 cm	117	5	122	
		35.30%	27.80%	35.00%	
Weight	60 kg or less	114	7	121	0.265
		34.40%	38.90%	34.70%	
	61 kg to 75 kg	103	8	111	
		31.10%	44.40%	31.80%	
	76 kg or more	114	3	117	
		34.40%	16.70%	33.50%	
Nationality	Saudi	245	17	262	0.051
		74.00%	94.40%	75.10%	
	Non-Saudi	86	1	87	
		26.00%	5.60%	24.90%	
Educational level	Primary school	2	0	2	0.001
		0.60%	0.00%	0.60%	
	Middle school	10	0	10	
		3.00%	0.00%	2.90%	
	High school	64	5	69	
		19.30%	27.80%	19.80%	
	Bachelor’s or higher	255	12	267	
		77.00%	66.70%	76.50%	
	Illiterate	0	1	1	
		0.00%	5.60%	0.30%	
Marital status	Single	217	13	230	0.769
		65.60%	72.20%	65.90%	
	Married	109	5	114	
		32.90%	27.80%	32.70%	
	Divorced or widowed	5	0	5	
		1.50%	0.00%	1.40%	

* *p* value was considered significant if ≤0.05.

## Data Availability

The original contributions presented in this study are included in the article/Supplementary Material. Further inquiries can be directed to the corresponding author.

## Supplementary Materials

The following supporting information can be downloaded at https://www.mdpi.com/article/10.3390/healthcare13222907/s1: Table S1: STOBE Checklist.

## Appendix A

**Table A1:** Questionnaire.

Biographical Data		
Age		
Age: ____		
Gender		
o Male	ο Female	
If you’re a female, are you pregnant right now?		
o Yes	ο No	
Height (in centimeters)		
Height: ____		
Weight (in kilograms)		
Weight: ____		
Are you a Saudi?		
o Yes	ο No	
Region of residence in Saudi Arabia:		
o Riyadh	ο Qassim	ο Eastern Province
ο Mecca	ο Madina	o Hail
ο Al-Bahah	ο Asir	ο Jazan
ο Najran	o Al-Jouf	ο Tabouk
ο Northern Borders		
Educational status?		
o Primary	ο Middle	ο High school
ο Bachelor’s or above		
Social status?		
o Single	ο Married	
Second section		
Do you suffer from any disease that causes dizziness or vertigo?		
o Yes	ο No	
If the answer was yes, please mention the disease: ______		
Do you suffer from dizziness or vertigo while being in a moving vehicle?		
o Yes	ο No	
If the answer was yes, please mention the severity of the dizziness or vertigo		
o Mild	ο Moderate	ο Severe
ο Unbearable		
Do you suffer from any other symptoms in addition to dizziness or vertigo?		
o Abdominal pain or discomfort	ο Nausea	ο Vomiting
ο Other:		
Do you have fear or anxiety while being in a moving vehicle?		
o Yes	ο No	
Do you suffer from headaches?		
o No	ο General Headache	ο Tension Headache
ο Migraine		
Do you suffer from any chronic illnesses?		
o No	ο Diabetes	ο Hypertension
ο Dyslipidemia	ο Asthma	o Others:
Have you had an infection in the past few days?		
o Yes	ο No	
If the answer was yes, please mention the type of infection _____		
Do you use any medications on a regular basis?		
o Yes	ο No	
If the answer was yes, please mention the medication and the reason for using the medication ______		
How many times have you used Riyadh’s Metro?		
o This is my first time	ο 2–3 times	ο More than 3 times
o Feeling of unbalance	ο None of the following	
Third section MSSQ Criteria and severity scale		
How do you spend your time while riding the metro?		
o Reading	ο Looking through the window	ο On your phone
o Talking with someone	ο Resting	ο Nothing
ο Other:		
Have you felt any of the following after your trip?		
o Sweating	ο Headache	ο Feeling hot
ο Change of skin color	o Mouth watering	ο Drowsiness
ο Dizziness	ο Nausea	ο Tiredness
o Vomiting	ο Burping	ο Abdominal cramps
How did you feel in general during your trip?		
o Felt well	ο Slight discomfort	ο Unwell
ο Felt ill		
If you felt that you’re not well, when did it happen?		
o When you got on the metro	ο When the metro started moving	o Sometime during the trip
ο At the end of the trip		
Has this occurred before?		
o Yes	ο No	
Did it get worse when you were looking through the window?		
o Yes	ο No	
Did it get worse when you noticed that you were moving in the opposite direction?		
o Yes	ο No	
Did you take something before or during the trip to feel better?		
o No	ο Gum	ο Ginger drink
ο Other		
When was the last time you ate?		
o During the trip	ο Just before the trip	ο Before 2–4 h
o Before more than 4 h		
What was it that you ate?		
o Full meal	ο Snack	ο Sweets
